# Cyclo-(l-Phe-l-Pro), a Quorum-Sensing Signal of Vibrio vulnificus, Induces Expression of Hydroperoxidase through a ToxR-LeuO-HU-RpoS Signaling Pathway To Confer Resistance against Oxidative Stress

**DOI:** 10.1128/IAI.00932-17

**Published:** 2018-08-22

**Authors:** In Hwang Kim, So-Yeon Kim, Na-Young Park, Yancheng Wen, Keun-Woo Lee, So-Young Yoon, Haneul Jie, Kyu-Ho Lee, Kun-Soo Kim

**Affiliations:** a Department of Life Sciences, Sogang University, Seoul, South Korea; b Research Institute for Basic Science, Sogang University, Seoul, South Korea; The University of Texas at Austin

**Keywords:** Vibrio vulnificus, cyclo-(l-Phe-l-Pro), quorum sensing, oxidative stress, hydroperoxidase, LeuO, RpoS, vHUαβ, HU

## Abstract

Vibrio vulnificus, an opportunistic human pathogen, produces cyclo-(l-Phe-l-Pro) (cFP), which serves as a signaling molecule controlling the ToxR-dependent expression of innate bacterial genes, and also as a virulence factor eliciting pathogenic effects on human cells by enhancing intracellular reactive oxygen species levels. We found that cFP facilitated the protection of V. vulnificus against hydrogen peroxide. At a concentration of 1 mM, cFP enhanced the level of the transcriptional regulator RpoS, which in turn induced expression of *katG*, encoding hydroperoxidase I, an enzyme that detoxifies H_2_O_2_ to overcome oxidative stress. We found that cFP upregulated the transcription of the histone-like proteins vHUα and vHUβ through the cFP-dependent regulator LeuO. LeuO binds directly to upstream regions of *vhuA* and *vhuB* to enhance transcription. vHUα and vHUβ then enhance the level of RpoS posttranscriptionally by stabilizing the mRNA. This cFP-mediated ToxR-LeuO-vHUαβ-RpoS pathway also upregulates genes known to be members of the RpoS regulon, suggesting that cFP acts as a cue for the signaling pathway responsible for both the RpoS and the LeuO regulons. Taken together, this study shows that cFP plays an important role as a virulence factor, as well as a signal for the protection of the cognate pathogen.

## Abstract (precis)

Vibrio vulnificus, an opportunistic human pathogen, produces cyclo-(l-Phe-l-Pro) (cFP), which serves as a signaling molecule controlling the ToxR-dependent expression of innate bacterial genes, and also as a virulence factor eliciting pathogenic effects on human cells by enhancing intracellular reactive oxygen species levels. We found that cFP facilitated the protection of V. vulnificus against hydrogen peroxide.

## INTRODUCTION

When infecting a host, bacterial pathogens encounter harsh environmental hazards, such as host defense systems, restricted access to nutrients, and oxidative stress. Generally, these conditions have a negative effect on bacterial proliferation and survival within the host. To overcome such challenges, bacteria have evolved various defense mechanisms (1). Many of these defense mechanisms are regulated by the alternative sigma factor RpoS (σ^S^), which is the master regulator of the general stress response (2, 3). RpoS is conserved among the gammaproteobacteria and is involved in the regulation of both virulence factors and stress responses in pathogenic bacteria, such as Vibrio vulnificus (4–7), a Gram-negative opportunistic pathogenic bacterium that causes septicemia and wound infections in humans (8–11). An RpoS-deficient mutant of V. vulnificus does not survive well under diverse environmental stresses, including exposure to hydrogen peroxide (H_2_O_2_), hyperosmolarity, and acidic conditions (7).

Cyclic dipeptides, also known as 2,5-diketopiperazines (DKPs), are naturally produced by animals, plants, fungi, and bacteria (12, 13). Most DKPs originate as by-products of fermentation or food processing (12), and there are also numerous endogenous DKPs that are produced by both animals and plants (12, 13). It has been reported that DKPs have antiviral, antibacterial, and antitumor activities (14–20). Cyclo-(l-phenylalanine-l-proline) (cFP) is produced by Vibrio spp. and is known to be a quorum-sensing signal that modulates the expression of the outer membrane protein OmpU in a ToxR-dependent manner (21–23). ToxR is an inner membrane protein present in pathogenic Vibrio spp. (24). In response to environmental signals that are not well understood, ToxR activates the ToxT regulon, stimulating expression of *ctxAB* (cholera toxin), TCP (the toxin-coregulated pilus), *ompT* (outer membrane porin), and accessory colonization factor (ACF), a protein that has been shown to repress the type VI secretion system in Vibrio cholerae (24–27). OmpU is associated with Vibrio sp. pathogenicity, conferring resistance to antibacterial peptides and bile acid, as well as aiding attachment to host cells (28–30).

Recently, it has been reported that cFP from V. vulnificus affects the NF-κB pathway in lipopolysaccharide (LPS)-stimulated monocyte/macrophage cell lines and also induces DNA double-strand breaks in a human intestinal cell line by increasing intracellular reactive oxygen species (ROS) (31, 32). ROS molecules generated in the host may harm the invading bacterial pathogen, and therefore, the pathogen must employ various means to detoxify these molecules (33). One of these mechanisms involves the enzyme KatG (hydroperoxidase I), which detoxifies H_2_O_2_ by converting it to H_2_O and O_2_ (34). We hypothesized that cFP may also be important in the response to ROS, and we investigated the protective effects of cFP in V. vulnificus, specifically through the expression of KatG. Our findings showed that cFP induces the expression of genes associated with detoxification of ROS via the master regulator RpoS, and we further demonstrate a molecular mechanism underlying the regulation of this process.

## RESULTS

### cFP facilitates the survival of V. vulnificus under H_2_O_2_-induced oxidative stress conditions.

**FIG 1:**
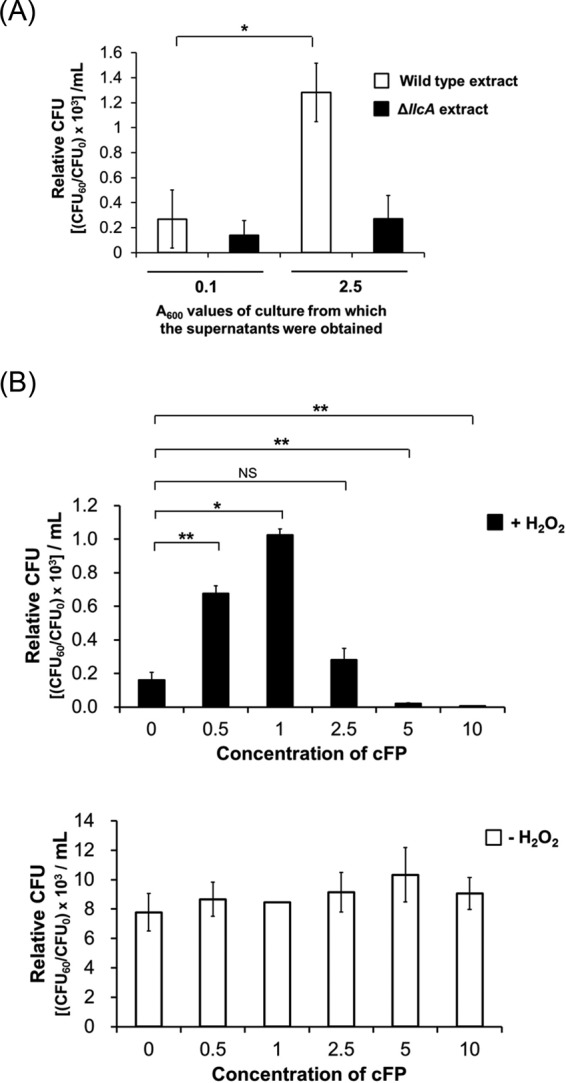
Effect of cFP on survival of V. vulnificus under H_2_O_2_-induced oxidative stress conditions. (A) Culture supernatant from wild-type V. vulnificus cells at stationary phase contributes to survival of V. vulnificus under oxidative stress. Wild-type V. vulnificus cells were treated with an ethyl acetate extract of supernatant from wild-type V. vulnificus or Δ*llcA* isotype cultures grown in LB broth to exponential phase (*A*_600_ = 0.1) or stationary phase (*A*_600_ = 2.5), and 625 μM H_2_O_2_ was added to each sample. Survival of cells was assessed by measuring CFU. (B) cFP at 1 mM increases survivability of V. vulnificus under H_2_O_2_-induced oxidative stress. Wild-type V. vulnificus cells grown in the presence of different concentrations of cFP were treated with 625 μM H_2_O_2_ (solid bars) or no H_2_O_2_ treatment (open bars). Cell survival was then assessed by measuring CFU. CFU were expressed as the CFU_60_/CFU_0_ ratio. CFU_0_ and CFU_60_ are CFU per milliliter at 0 min and at 60 min, respectively, after treatment or not with H_2_O_2_. The error bars denote standard deviations of the results of three independent experiments (**, *P* < 0.005; *, *P* < 0.05; NS, not significant).

A recent study showed that cFP produced by V. vulnificus increases the intracellular levels of ROS in human cell lines, resulting in apoptosis (31, 32). This report led us to hypothesize that cFP may be associated with the response of V. vulnificus to oxidative stress, as well. To test this, we measured the survival rate of V. vulnificus under H_2_O_2_-induced stress conditions after supplementing cells with cFP that had been extracted from culture supernatants of either wild-type V. vulnificus (strain MO6-24/O) or V. vulnificus Δ*llc*, a mutant MO6-24/O strain defective in cFP production (32) (Fig. 1A). cFP can be readily extracted from culture supernatant using ethyl acetate (21). Supplementing V. vulnificus with extracts from a stationary-phase culture supernatant resulted in a significantly increased survival rate. However, survival was not improved when cells were treated with extracts from either MO6-24/O grown to early exponential phase or from the Δ*llc* mutant. These results suggest that the cFP produced in wild-type V. vulnificus while in stationary phase (21) enhances the survival of V. vulnificus under oxidative stress. We also assessed the survival of V. vulnificus in the presence of H_2_O_2_-induced ROS by supplementing with various concentrations (0 to 10 mM) of chemically synthesized cFP. As shown in Fig. 1B, treatment of peroxide-stressed wild-type V. vulnificus with up to 1 mM cFP, representing the physiological concentration of cFP in a culture supernatant of V. vulnificus at stationary phase (21), increased survival in a concentration-dependent manner. The relative CFU numbers in the presence of 1 mM cFP were at least 5-fold higher than without cFP (Fig. 1B, left graph). However, when cFP was added at higher concentrations, survivability decreased drastically, and at 10 mM, CFU were barely detectable. In contrast, under non-oxidative stress conditions (no H_2_O_2_ treatment), the survival of V. vulnificus was not influenced by cFP even at concentrations of 5 or 10 mM (Fig. 1B, right graph).

A previous study demonstrated that some DKPs, especially those derived from the cyclization of an amino acid with leucine, exhibit antioxidant activity due to their innate ability to scavenge radicals (35). We considered the possibility that enhancement of the survival of H_2_O_2_-treated V. vulnificus cells by cFP may also be due to the capability of cFP itself to scavenge radicals. We tested this by performing a DPPH (2,2-diphenyl-1-picrylhydrazyl) radical-scavenging assay, as described in Materials and Methods. Using l-ascorbic acid as a positive control (36), the DPPH radical-scavenging activity was measured in the presence of several concentrations of cFP. While l-ascorbic acid showed antioxidant activity in a concentration-dependent manner, cFP did not exhibit any significant antioxidant activity (see Fig. S1 in the supplemental material). This result suggested that cFP cannot reduce intracellular ROS levels through direct molecular interactions; rather, it may enhance the survivability of V. vulnificus by affecting signaling pathways involved in oxidation stress survival.

### cFP induces the expression of *katG* via RpoS.

**FIG 2:**
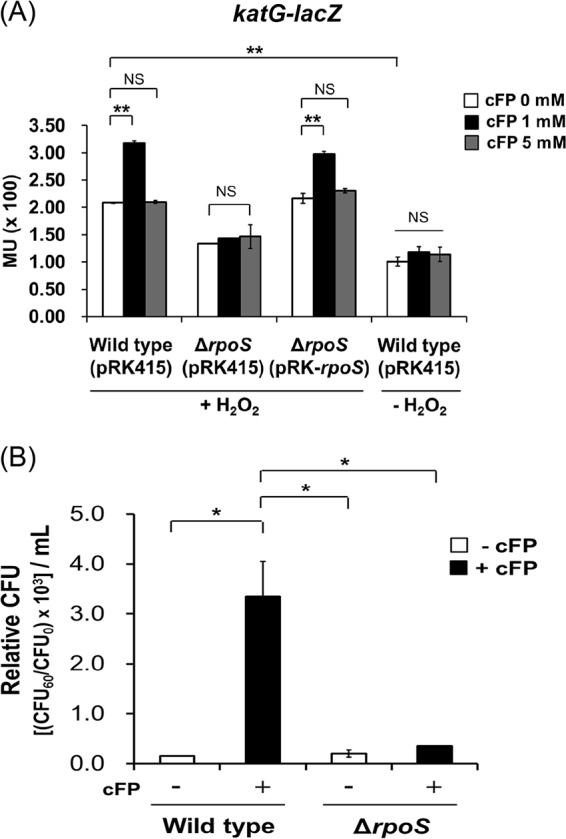
Expression of *katG* is influenced by cFP and *rpoS*. (A) Transcription levels of *katG* as measured by β-galactosidase activities from wild-type V. vulnificus MO6-24/O [Wild type (pRK415)]; an isotype, KPR101, with *rpoS* deleted [Δ*rpoS* (pRK415)]; and KPR101 complemented with the *rpoS* gene [Δ*rpoS* (pRK-*rpoS*)] harboring pMZtc-katG at 0 mM, 1 mM, and 5 mM cFP under H_2_O_2_-induced oxidative conditions or without H_2_O_2_. H_2_O_2_ (625 μM) was added during the exponential growth phase of the cells (*A*_600_, ∼0.1) in AB broth. The data are the average values of the results of three independent experiments, and the error bars denote standard deviations. MU, Miller units. (B) Effect of 1 mM cFP on the survival of V. vulnificus MO6-24/O (Wild type) and KPR101 (Δ*rpoS*) strains in the presence of 625 μM H_2_O_2_. The error bars denote standard deviations of three independent experiments (**, *P* < 0.005; *, *P* < 0.05; NS, not significant).

KatG (hydroperoxidase I) is a key enzyme responsible for H_2_O_2_ detoxification, and it has been reported that expression of *katG* is regulated by RpoS in V. vulnificus (34). We considered the possibility that cFP exerts an antioxidant effect by altering the expression of *katG*. To test this, we measured the effects of various cFP concentrations on *katG* expression levels using a *katG-lacZ* transcriptional reporter fusion (Fig. 2A) under 625 μM H_2_O_2_-induced oxidative conditions. Expression of *katG* in wild-type V. vulnificus MO6-24/O was about 1.7 times higher in the presence of 1 mM cFP than with 0 and 5 mM cFP. In contrast, in KPR101 (34), a V. vulnificus isotype with *rpoS* deleted, varying cFP concentrations had no effect. Introducing an *rpoS* clone back into the mutant restored the response to cFP. In the absence of H_2_O_2_, the expression of *katG* in the wild-type strain was not affected by cFP and was about half the level observed under H_2_O_2_-induced oxidative conditions, suggesting that H_2_O_2_ enhances *katG* expression, although to much less significant levels than cFP. We also quantitatively compared the survivability of MO6-24/O and KPR101 after treatment with 1 mM cFP, followed by 625 μM H_2_O_2_ (Fig. 2B). Consistent with the hypothesis that cFP affects *katG* transcription, cFP did not enhance survivability in KPR101, but 1 mM cFP dramatically increased the survivability of wild-type cells (Fig. 2B). These results indicate that cFP at 1 mM activates KatG expression under oxidative stress conditions via an RpoS-regulated pathway and that this activation is responsible for the increased survivability of V. vulnificus under oxidative stress conditions induced by H_2_O_2_.

### cFP activates the expression of *rpoS* at the posttranscriptional level.

**FIG 3:**
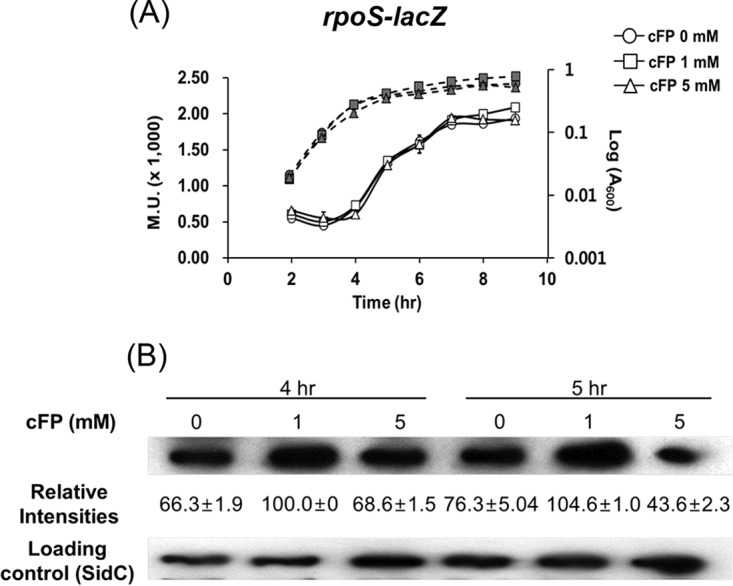
RpoS expression is enhanced at the posttranscriptional level in the presence of 1 mM cFP. (A) Transcription levels of *rpoS* as measured by β-galactosidase activities (solid lines) and growth curves (dashed lines) of V. vulnificus MO6-24/O cultured in AB broth harboring pMZtc-rpoS (*rpoS-lacZ* fusion) and treated with 0 mM, 1 mM, and 5 mM cFP. The data are the averages of the results of three independent experiments, and the error bars denote standard deviations. M.U., Miller units. (B) Western hybridization using polyclonal antisera against RpoS of total protein extracts from V. vulnificus MO6-24/O cultured in AB broth treated in early stationary phase with cFP at 0 mM, 1 mM, and 5 mM. (From left) Lane 1, treated with DMSO (dimethyl sulfoxide) (no cFP); lane 2, treated with 1 mM cFP; lane 3, treated with 5 mM cFP; 40 μg of the total protein from each sample was loaded on the gel. For a loading control, antiserum against SidC (insulin-degrading enzyme) (59), a protein that is not modulated by cFP, was used. The relative intensities of the bands were measured using Multi Gauge ver. 3.0 (Fujifilm, Tokyo, Japan).

We assessed the effect of cFP on the level of *rpoS* transcription using an *rpoS-lacZ* transcriptional fusion (Fig. 3) and growing cells in AB (autoinducer bioassay) minimal medium. In AB minimal medium, even though growth of V. vulnificus is somewhat limited and cells reach stationary phase at an *A*_600_ of 0.2, intracellular biosynthesis of cFP is lower (unpublished data) and the effects of exogenously supplemented cFP are more evident. β-Galactosidase expression of the *rpoS-lacZ* transcriptional fusion in the presence of cFP at three different concentrations (0, 1, and 5 mM) did not differ significantly (Fig. 3A). RpoS is a central regulator of general stress responses and is controlled by various factors at the transcriptional, translational, and posttranslational levels (2, 37). We measured the levels of RpoS translation through Western hybridization of cell extracts at early stationary phase (*A*_600_ = 0.2) and mid-stationary phase (*A*_600_ = 0.3) using polyclonal rabbit antisera against purified RpoS. We observed about 1.5-fold higher expression levels of RpoS in the presence of 1 mM cFP than when cFP was added at 0 mM or 5 mM (Fig. 3B), indicating that the presence of cFP affected *rpoS* at a posttranscriptional or translational level.

### vHUαβ are involved in the enhancement of RpoS expression elicited by cFP.

**FIG 4:**
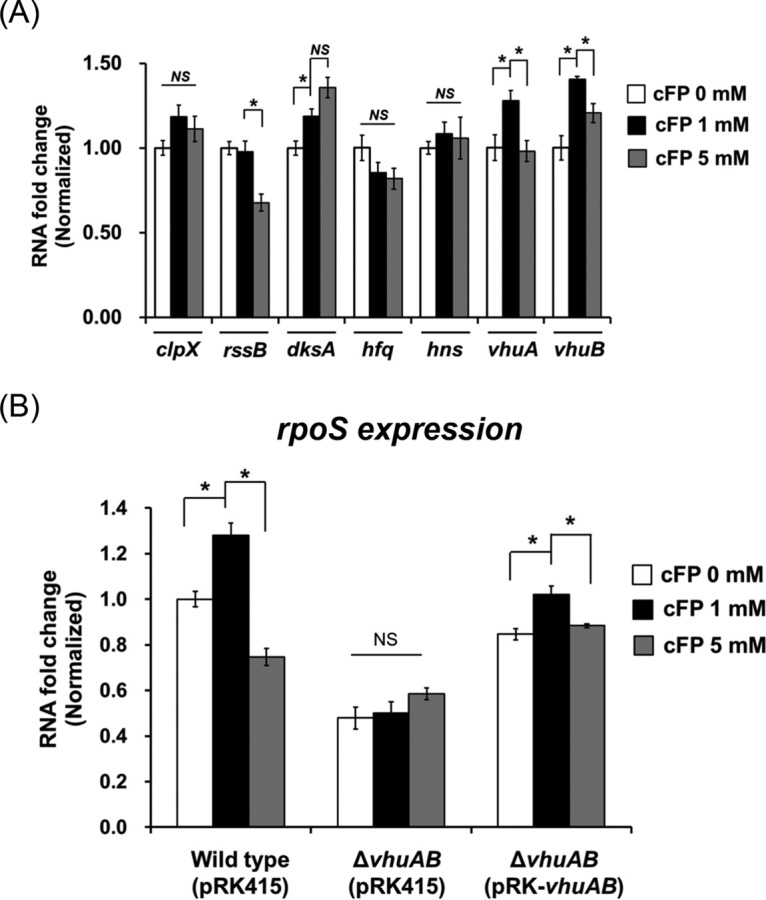
RpoS expression is regulated by cFP via vHUαβ. (A) The effect of cFP on the expression levels of *clpX*, *rssB*, *dksA*, *hfq*, *hns*, and *vhuAB* was assessed by qRT-PCR. RNA was isolated from wild-type V. vulnificus MO6-24/O cultured in AB broth and treated with 0 mM, 1 mM cFP, and 5 mM cFP at early stationary phase (*A*_600_, ∼0.2). (B) Expression levels of RpoS as measured by qRT-PCR. RNA samples were obtained from wild-type (pRK415), ΔvhuAB(pRK415), and ΔvhuAB(pRK-vhuAB) cultured in AB broth at early stationary phase (*A*_600_, ∼0.2). All the RNA levels were quantified using the comparative threshold cycle (ΔΔ*C_T_*) method, and RNA fold change values were normalized to the value for MO6-24/O without cFP (*, *P* < 0.05; NS, not significant).

To determine how cFP affects the expression of RpoS, we examined each of the various posttranscriptional and proteolysis factors associated with intracellular RpoS levels. It has been documented that expression of RpoS is stimulated or stabilized by HUαβ and Hfq and repressed by H-NS at the posttranscriptional level in other bacterial species (2, 37). It has also been previously reported that RpoS is degraded by ClpX, which in turn is regulated by RssB (3, 37). We assessed the effect of cFP on the expression of these various factors using quantitative real-time PCR (qRT-PCR) (Fig. 4A). The results showed that expression level patterns of *vhuA* and *vhuB* (Vibrio HUα and -β genes) overlapped with that of *katG*; *vhuA* and *vhuB* expression levels were significantly enhanced with 1 mM cFP and decreased with 5 mM cFP (Fig. 4A). Expression levels of *clpX*, *hfq*, and *hns* were not significantly affected by cFP. Expression of *rssB* was significantly decreased in the presence of 5 mM cFP but was not affected by 1 mM cFP. For *dksA*, expression levels were enhanced by the addition of 1 mM cFP and were even higher with 5 mM cFP. These results suggest that DksA, vHU*α*, and vHU*β* are all involved in the cFP-dependent enhanced expression of *rpoS* in V. vulnificus. Our previous transcriptomic analysis of genes affected by cFP in wild-type V. vulnificus showed that transcription levels of vHUα and -β were increased in the presence of cFP (38). Therefore, we focused on vHUα and -β, which showed a cFP-dependent expression pattern similar to that of RpoS, for further studies. qRT-PCR was used to compare levels of *rpoS* transcripts in wild-type V. vulnificus and an isotype with deletions of the *vhuAB* genes (Fig. 4B). The expression level of *rpoS* was higher in the presence of 1 mM cFP in wild-type cells. However, in the *vhuAB* deletion mutant, the basal level of RpoS expression without cFP was just half the wild-type level, and addition of cFP had no significant effect. Complementation of the *vhuAB* deletion mutant with exogenous *vhuAB* on a plasmid restored expression of *rpoS* when 1 mM cFP was supplemented. This suggests that in V. vulnificus, vHUαβ proteins are required for a basal level of *rpoS* expression in the absence of cFP and are required for cFP-mediated induction of *rpoS* expression.

### The cytoplasmic regulator LeuO is responsible for the cFP-dependent induction of *vhuAB* expression.

**FIG 5:**
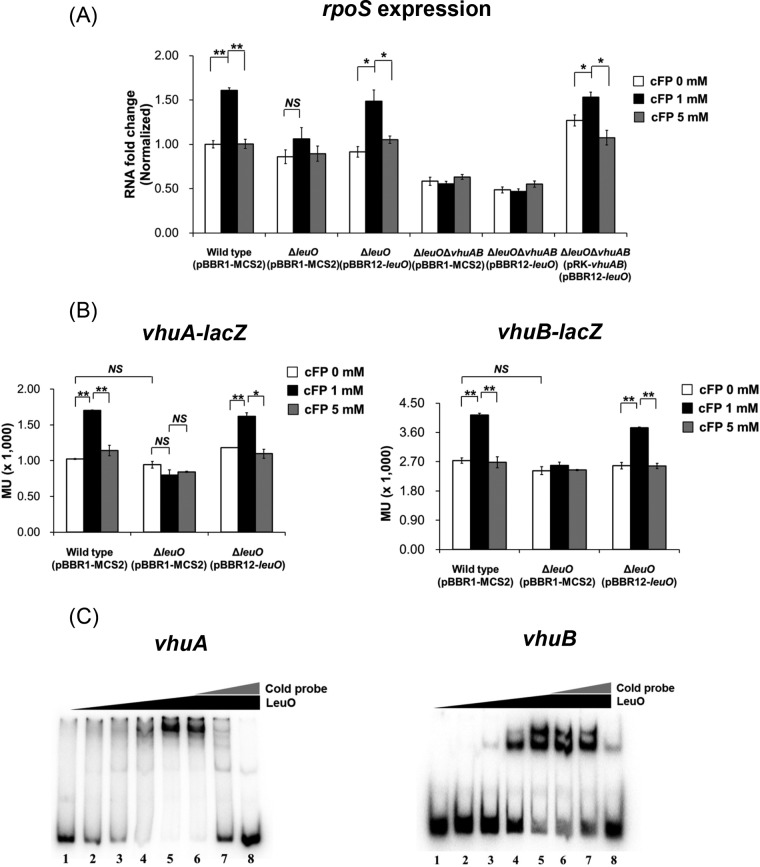
cFP enhances the expression of genes encoding subunits of a histone-like protein, vHUα and vHUβ, via LeuO. (A) Expression levels of *rpoS* as measured by qRT-PCR from wild-type V. vulnificus [Wild type (pBBR1-MCS2)], Δ*leuO*(pBBR1-MCS2), Δ*leuO*(pBBR1-leuO), Δ*leuO*Δ*vhuAB*(pRK415), and Δ*leuO*Δ*vhuAB*(pRK-*vhuAB*) cultured in AB broth at early stationary phase (*A*_600_, ∼0.2). (B) β-Galactosidase activities from wild-type (pBBR1-MCS2), Δ*leuO*(pBBR1-MCS2), and Δ*leuO*(pBBR1-*leuO*) strains harboring pMZtc-vhuA (left) or pMZtc-vhuB (right). Overnight cultures of V. vulnificus were subcultured into fresh LB broth supplemented with each concentration of cFP, and when the cells reached exponential phase (optical density [OD], 0.3 to 0.4), the cells were resubcultured in fresh AB broth. β-Galactosidase activities were measured as described in Materials and Methods. The error bars denote standard deviations of the results of three independent experiments. MU, Miller units. **, *P* < 0.005; *, *P* < 0.05; NS, not significant. (C) Binding of recombinant LeuO (rLeuO) to the upstream regions of *vhuA* (left) and *vhuB* (right) genes as determined by electrophoretic mobility shift assay. Ten nanograms of radiolabeled probes was incubated with increasing concentrations of LeuO. Lanes 1 to 5, LeuO concentrations of 0 nM, 10 nM, 20 nM, 40 nM, and 80 nM, respectively; lanes 6 to 8, 80 nM rLeuO with unlabeled probes as a competitor at 1 ng, 10 ng, and 100 ng, respectively.

The cytoplasmic LysR-type regulator LeuO is involved in the cFP signaling pathway in both V. vulnificus (our unpublished data) and V. cholerae (23). We used qRT-PCR to examine the possibility that LeuO is involved in the cFP-signaling regulation of *rpoS* by comparing transcription levels of *rpoS* in the wild type, a *leuO* deletion mutant, and a *leuO vhuAB* triple-deletion mutant (Fig. 5A). Deletion of *leuO* abolished the cFP-mediated induction of *rpoS*, and introduction of a *leuO* clone restored induction in the presence of 1 mM cFP. In a *leuO vhuAB* triple-deletion mutant, expression of *rpoS* was not induced by cFP, and *rpoS* expression levels were significantly lower than in the wild type. Introduction of either *leuO* or *vhuAB* independently into the triple mutant failed to restore cFP induction. However, when all three genes were reintroduced, *rpoS* expression was restored. These results suggested that LeuO and vHUαβ are both involved in cFP signaling regulation of *rpoS* expression, and this led us to hypothesize that cFP signaling is transduced to *vhuAB* through LeuO. To test this, the effect of LeuO on *vhuA* and *vhuB* transcription levels was quantitatively measured using *vhuA-lacZ* and *vhuB-lacZ* transcriptional fusions (Fig. 5B). Expression of β-galactosidase from each of these *lacZ* fusions in a wild-type strain increased by up to about 1.5-fold in the presence of 1 mM cFP. In contrast, in a *leuO* deletion mutant, transcription was not affected by cFP. Introduction of an exogenous *leuO* clone into the *leuO* deletion mutant restored the cFP-induced expression of *vhuA* and *vhuB*. LeuO is a LysR family transcriptional regulator that binds directly to the promoter of target genes (39). To determine whether the effect of LeuO on expression of vHUα and vHUβ is mediated by direct binding to *cis*-acting elements in upstream regions of the coding genes, gel shift assays were performed using a recombinant LeuO protein (39) and a DNA probe, including the upstream regions of *vhuA* and *vhuB* (Fig. 5C). LeuO binds to upstream regions of both *vhuA* and *vhuB* in a concentration-dependent manner. These results show that LeuO regulates expression of *vhuA* and *vhuB* by binding directly to their *cis*-acting elements in upstream regions.

### Expression of *leuO* is activated by cFP in a concentration-dependent manner.

**FIG 6:**
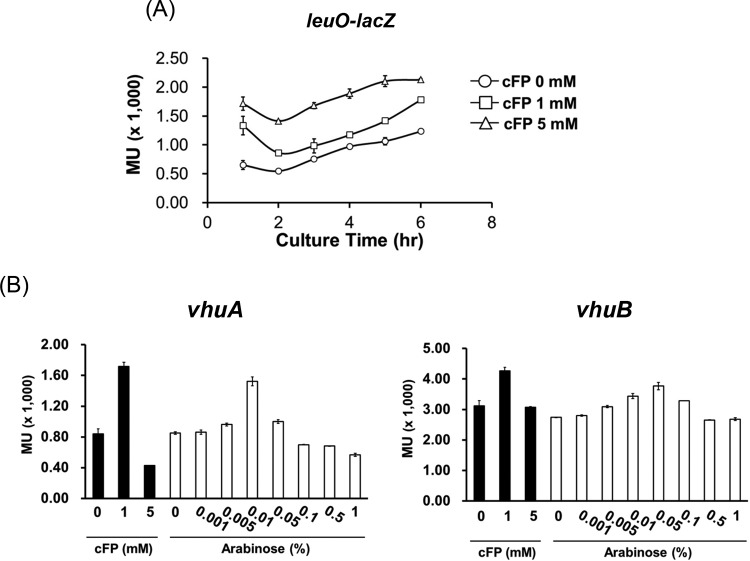
Transcriptional levels of *leuO* under various concentrations of cFP and transcriptional levels of *vhuA* and *vhuB* modulated by LeuO differentially expressed in the arabinose induction system. (A) Transcription levels of LeuO as measured by β-galactosidase activities of V. vulnificus MO6-24/O harboring pMZtc-leuO cultured in AB broth and treated with 0 mM, 1 mM, or 5 mM cFP. (B) Transcription levels of *vhuA* (left) and *vhuB* (right) as measured by β-galactosidase activities from MO6-24/O(pBBR1-MCS2) harboring pMZtc-vhuA or pMZtc-vhuB and from Δ*leuO*(pBBR12-leuO-ara) harboring pMZtc-vhuA or pMZtc-vhuB cultured in AB broth. The culture conditions are described in Materials and Methods. To induce the expression of LeuO by arabinose, the culture was split into seven aliquots when the *A*_600_ reached 0.1, and then various concentrations of arabinose (0, 0.005, 0.01, 0.05, 0.1, 0.5, and 1%) were added. The error bars denote standard deviations of the results of three independent experiments. MU, Miller units.

The above-described results suggest that LeuO regulates the expression of *vhuA* and *vhuB* and that this expression is induced by 1 mM cFP, but not with 5 mM cFP. Therefore, we predicted that *leuO* expression would follow a similar pattern. We used a *leuO-lacZ* transcriptional reporter fusion to measure expression of *leuO* in cells grown in media containing cFP at 0, 1, and 5 mM. Surprisingly, *leuO* was expressed at higher levels with 5 mM cFP than it was with 1 mM (Fig. 6A). We then measured the transcription of *vhuA* and *vhuB* using *lacZ* fusions in a Δ*leuO* strain harboring an arabinose-inducible *leuO* overexpression vector (Fig. 6B). The level of *vhuA* transcription increased in proportion to the arabinose concentration up to 0.01% arabinose. At higher arabinose concentrations, however, *vhuA* transcription levels started to decrease. The expression of *vhuB* followed a similar pattern. These results suggest that expression of *vhuA* and *vhuB* reaches a maximal level at a particular intracellular concentration of LeuO and then decreases at higher LeuO concentrations.

### RpoS mRNA is stabilized by LeuO-vHU*αβ* signal transduction elicited by cFP.

**FIG 7:**
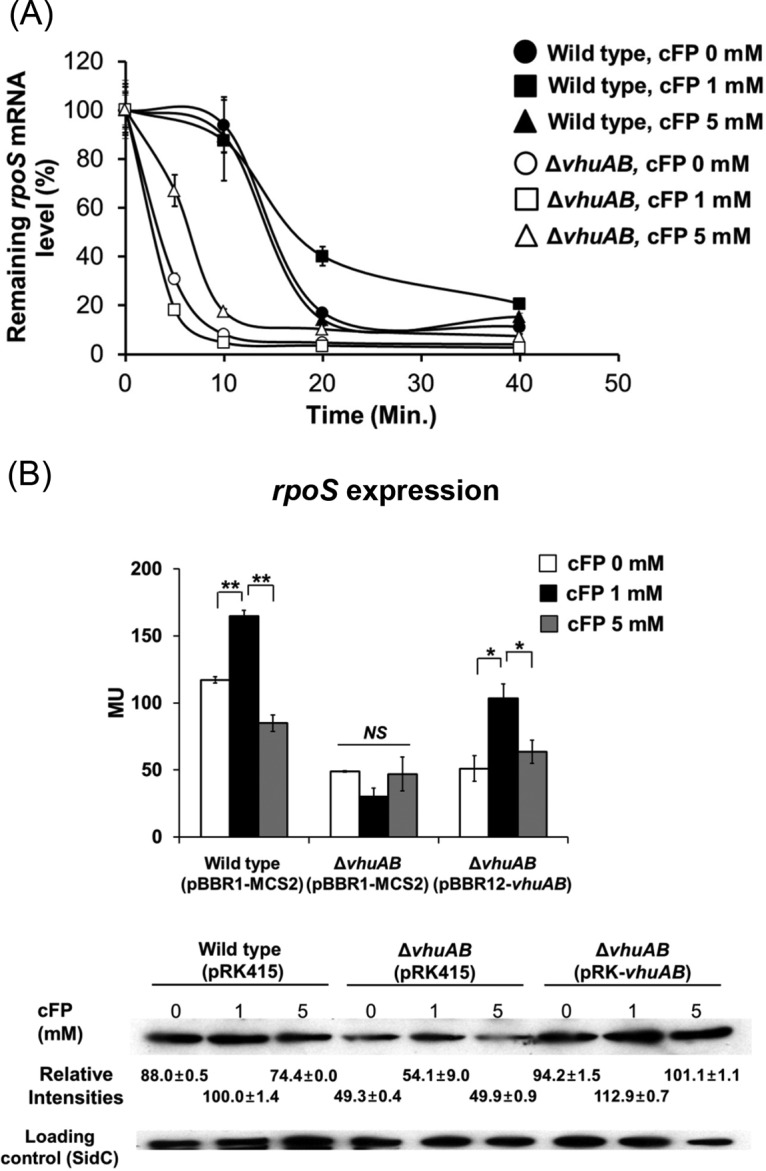
Enhancement of stability of RpoS mRNA by cFP. (A) The relative mRNA level of *rpoS* after rifampin treatment was measured by qRT-PCR. Wild-type V. vulnificus MO6-24/O and the Δ*vhuAB* deletion mutant were treated with rifampin when growth in AB broth reached an *A*_600_ of 0.2. At 0, 5, 10, 20, and 40 min after the treatment, RNA was isolated from cells for qRT-PCR analysis. Half-lives were calculated using GraphPad Prism 5. The data are average values from three independent samples, and the error bars denote standard deviations. (B) Translation level of *rpoS* as measured by β-galactosidase activity and Western hybridization using antiserum against RpoS of V. vulnificus wild type (pBBR1-MCS2), Δ*vhuAB*(pBBR1-MCS2), and Δ*vhuAB*(pBBR12-*vhuAB*) harboring pRZtl-rpoS cultured in AB broth. Measurements of β-galactosidase activities and Western hybridization were performed as described in Materials and Methods. The error bars denote standard deviations of the results of three independent experiments. MU, Miller units. **, *P* < 0.005; *, *P* < 0.05; NS, not significant).

The above-mentioned result suggests that regulation of RpoS expression by cFP is at the posttranscriptional level. We assessed the stability of the *rpoS* mRNA after treatment with cFP in both wild-type V. vulnificus and a *vhuAB* deletion mutant by treating cells with cFP and then adding rifampin to interrupt transcription. The relative *rpoS* mRNA levels in each of these strains were assessed by qRT-PCR in a time course experiment (Fig. 7A). The half-life of *rpoS* mRNA in wild-type V. vulnificus grown in the presence of 1 mM cFP was about 29.8 min, and those of cells grown either without cFP or with 5 mM cFP were 16.0 and 16.5 min, respectively. In a *vhuAB* deletion mutant, the half-life of *rpoS* mRNA was much shorter, at 2.7 (0 mM cFP), 1.9 (1 mM cFP), and 5.2 (5 mM cFP) min. RpoS protein levels were higher in wild-type V. vulnificus after treatment with cFP, but in the *vhuAB* deletion mutant, even though overall levels were lower, there was no increase in the presence of 1 mM cFP (Fig. 7B). Complementation of the *vhuAB* deletion mutant with exogenous *vhuAB* on a plasmid restored expression of *rpoS* when supplemented with 1 mM cFP. These results indicate that 1 mM cFP enhances RpoS expression at the posttranscriptional level, most likely by enhancing mRNA stability through the activity of vHUαβ.

### cFP affects the expression of the RpoS regulon.

**FIG 8:**
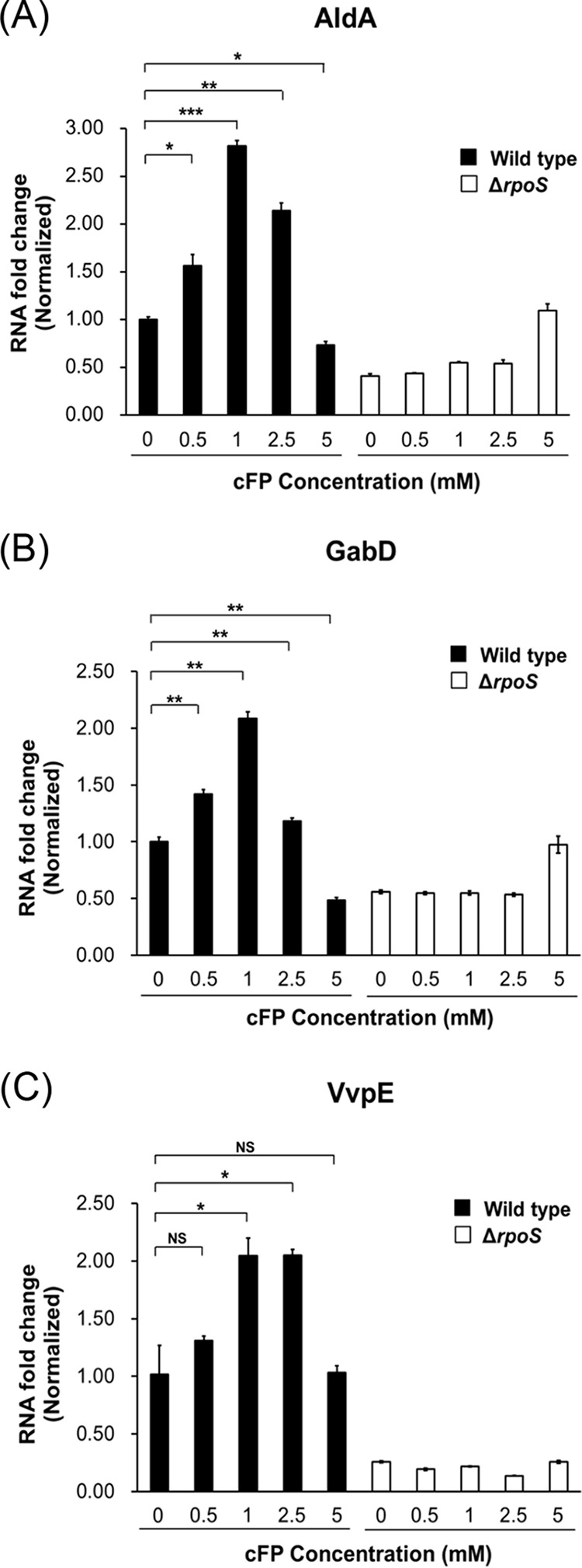
Transcription of the RpoS-inducing genes *aldA*, *gabD*, and *vvpE* is also induced by 1 mM cFP. A comparison of the transcription levels of *aldA* (A), *gabD* (B), and *vvpE* (C) in wild-type V. vulnificus MO6-24/O and KPR101 (Δ*rpoS*) cultured in AB broth is shown. Cells were treated with cFP (0, 1, or 5 mM), and RNA samples obtained at early stationary phase (*A*_600_, ∼0.2) were analyzed by qRT-PCR using the primers shown in Table S2 in the supplemental material. Overnight cultures were subcultured in AB minimal media supplemented with cFP (0, 1, and 5 mM). RNA levels were quantified using the ΔΔ*C_T_* method, and the RNA fold change was normalized to the value for MO6-24/O cultured with 0 mM cFP (DMSO buffer only). The data are average values from three independent samples, and the error bars denote the standard deviations (***, *P* < 0.001; **, *P* < 0.005; *, *P* < 0.05; NS, not significant).

We showed that the expression of *katG* is increased in the presence of 1 mM cFP due to regulation of *rpoS* expression. The next question was whether cFP influenced the expression of other genes in V. vulnificus previously reported to be regulated by RpoS, including *aldA*, *gabD*, and *vvpE* (40, 41). These genes encode an aldehyde dehydrogenase, a succinate-semialdehyde dehydrogenase, and a metalloprotease, respectively. Expression levels of the genes in wild-type V. vulnificus MO6-24/O and in the *rpoS* deletion isotype strain KPR101 after treatment with various concentrations of cFP were measured using qRT-PCR (Fig. 8). In wild-type cells, the expression of each gene was enhanced in the presence of 1 mM cFP. However, when 5 mM cFP was added, expression was not significantly different from that in cells with no cFP treatment. In contrast, in KPR101 cells, expression of the three genes did not increase significantly upon the addition of 1 mM cFP, suggesting that RpoS is required. To summarize, these results indicate that 1 mM cFP led to the increased expression of genes in the RpoS regulon.

### Effects of cFP on the expression of *leuO*, *vhuAB*, and *rpoS* in V. cholerae and V. parahaemolyticus.

**FIG 9:**
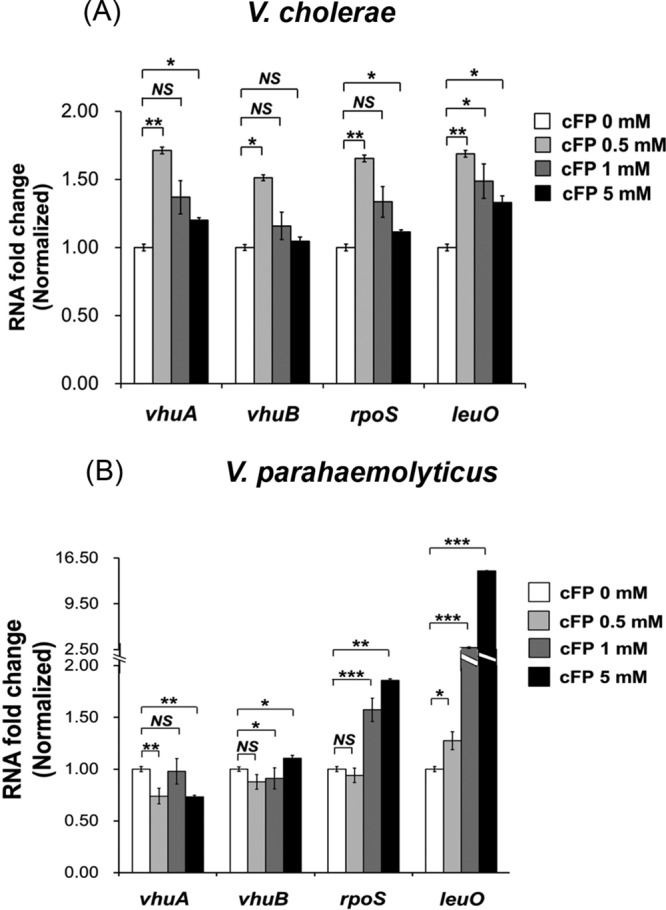
Effect of cFP on expression of genes encoding histone-like protein HUα and HUβ subunits, RpoS, and LeuO in V. cholerae and V. parahaemolyticus. Shown is a comparison of transcription levels of *vhuA*, *vhuB*, *rpoS*, and *leuO* treated with cFP in wild-type V. cholerae (A) and V. parahaemolyticus (B) at early stationary phase (*A*_600_, ∼0.2) by qRT-PCR using the primers shown in Table S2 in the supplemental material. Overnight cultures were subcultured in LB medium and then subcultured in AB minimal media containing cFP (0, 0.5, 1, and 5 mM). RNA levels were quantified using the ΔΔ*C_T_* method, and the RNA fold change of each gene was normalized to the value for cells cultured with 0 mM cFP (DMSO only). The data are average values from three independent samples, and the error bars denote standard deviations (***, *P* < 0.001; **, *P* < 0.005; *, *P* < 0.05; NS, not significant).

V. cholerae and Vibrio parahaemolyticus also produce cFP (21). In these related human pathogens, cFP modulates genes through ToxR (21), suggesting that, as in V. vulnificus, cFP may trigger expression of *leuO*, *vhuAB*, and *rpoS*. To test this assumption, the expression of each of the three genes in the presence of cFP at varying concentrations was assessed using qRT-PCR (Fig. 9). The expression pattern in V. cholerae was similar to that seen previously for V. vulnificus in that each of the three genes was expressed at the highest level in the presence of 0.5 mM cFP and then was lower at higher concentrations of cFP. However, a different pattern was observed for V. parahaemolyticus in that cFP had minimal effect on the expression of *vhuAB*, and expression of both *rpoS* and *leuO* increased gradually in a cFP-dependent manner (up to 5 mM cFP).

## DISCUSSION

In this study, we showed that in V. vulnificus, the diketopiperazine compound cFP facilitates survival under H_2_O_2_ oxidative stress, not by direct antioxidant activity, but by upregulating hydroperoxidase through a complex signal transduction pathway that includes hierarchical regulatory components. It has been suggested that in wild-type V. vulnificus (MO6-24/O), which lacks the canonical homoserine lactone signal, cFP acts as a quorum-sensing signal in addition to autoinducer-2 (AI-2) (21, 22). This study, along with our previous study (28), describes the first example of a quorum-sensing signaling molecule that has a distinctly different second role as a direct virulence factor and an inducer of pathways that protect the pathogen from damage induced by the signal molecule in the host.

The direct effector of this cFP-associated antioxidant stress response is the hydroperoxidase KatG, while the regulator of this function is the alternative sigma factor RpoS (Fig. 2). RpoS is a master regulator of the general stress response in many bacteria, and its intracellular concentration is regulated by many factors at transcriptional, translational, and posttranslational levels (2, 3, 37). RpoS is regulated at the transcriptional level by factors including ArcAB, Crp, and (p)ppGpp (2, 3); at the translational level by factors including Hfq and small RNAs (e.g., DsrA, RprA, and ArcZ) (2, 37); and at the regulatory level by HN-S, OxyS, HUαβ, DksA, CsdA, and CspCE (2, 3). RNase III is important for the stability of RpoS mRNA (2) and proteolytic processing, and along with numerous factors, such as ClpXP, RssB, and the antiadaptors IraPMD (required for the stability of RpoS by interfering with RssB activity), controls RpoS protein levels (3). Some of these factors enhance intracellular RpoS levels, while others reduce it. From our previous transcriptomic study (38), we identified factors affected by cFP in such a way that an increase in intracellular RpoS would be expected. Among the factors that influence RpoS, *oxyS*, *arcZS*, *dsrA*, and *iraPDM* are not found in the genome of V. vulnificus. In the presence of cFP, expression levels of *relA* and *arcB* and the gene encoding RNase III are not enhanced. However, expression levels of *vhuAB* and *dksA*, factors known to be required for the stabilization of RpoS mRNA, were higher in the presence of cFP. In the current study, qRT-PCR experiments confirmed that cFP induced the expression of these genes (Fig. 4A). DksA is known to activate translation of *rpoS* indirectly via Hfq (42). However, in our results, expression of Hfq was not significantly affected by cFP. Particularly interesting was the effect of cFP on the expression patterns of *vhuAB*, which followed a trend similar to that of *katG*. The observed results suggested that the histone-like proteins HUαβ are the regulatory components directly responsible for cFP-dependent regulation of RpoS, and hence, we focused on these factors for further studies.

The histone-like proteins HUαβ are known to be major components of nucleoid-associated proteins and are conserved in most bacteria (43). HUαβ can bind to DNA or RNA by recognizing a specific structure (44). Although functions associated with DNA binding have been studied extensively (45–47), a role in RNA binding is not understood. HUαβ have been shown to bind to *rpoS* and *dsrA* RNAs and some noncoding RNAs (44, 48, 49). In Escherichia coli, HUαβ bind to *rpoS* mRNA specifically and stimulate translation but do not affect stability (48). Therefore, it has been suggested that HUαβ modify the RNA secondary structure to facilitate ribosome binding or, alternatively, modulate binding of other factors, such as Hfq or H-NS, or both. However, vHUαβ appear to affect the *rpoS* mRNA stability in V. vulnificus (Fig. 4B and 7A). How these proteins affect translational regulation of RpoS in V. vulnificus remains to be elucidated.

Currently, it is not clear why we observed lower expression of *katG* in the presence of 5 mM cFP than with 1 mM cFP (Fig. 2). The expression of *leuO* is increased by cFP in a concentration-dependent manner (Fig. 6A). However, the expression of *vhuAB*, which is just downstream of LeuO in the signaling pathway, was lower at 5 mM cFP, and increased levels of LeuO resulted in a decrease in *vhuAB* above a certain concentration of cFP, suggesting that LeuO induces the expression of *vhuAB* only to a certain level and that the effect is abolished above that level. A similar LeuO induction pattern was observed in Salmonella enterica (50, 51). Multiple binding sites for LeuO in the upstream regions of *vhuAB*, as suggested by electrophoretic mobility shift assays (Fig. 5C), are thought to be important for this regulation. Higher concentrations of cFP could be toxic to V. vulnificus, and cFP, together with H_2_O_2_, may be synergistically harmful to the pathogen (Fig. 1B). However, the physiological concentration of cFP in a culture supernatant of V. vulnificus at stationary phase is 0.7 to 1.0 mM (21), which, as shown in this study, is optimal for conferring resistance to H_2_O_2_. It is unlikely that V. vulnificus encounters a higher concentration of cFP under conditions that prevail in natural habitats.

The culture conditions used for growth of V. vulnificus in this study may not precisely mimic the conditions the pathogen encounters in a host, and the precise concentrations of cFP produced by the pathogen while in the host are unknown. However, our previous studies showed that infection of human cells with V. vulnificus led to physiological changes that were similar to those we observed when cells were treated with 1 mM exogenous synthetic cFP (33, 34), suggesting that our *in vitro* conditions accurately mimic the cFP produced by V. vulnificus. Nevertheless, further study is necessary to determine whether the results from this study represent biological events that occur during pathogen infection.

We extended our study to the related important pathogens V. cholerae, which is the causative agent of cholera, and V. parahaemolyticus, which causes gastrointestinal illness in humans. The effect of cFP on V. cholerae was similar to that observed for V. vulnificus. In V. cholerae, LeuO is upregulated by cFP (23) and then represses *aphA*, a gene encoding the first in a cascade of regulatory proteins that eventually affects levels of both cholera toxin and toxin-related pilus. It is likely that the pathogen also harbors a cFP signaling pathway for the regulation of catalase similar to that of V. vulnificus. In contrast, V. parahaemolyticus employs a cFP-associated signal transduction pathway at least partly distinct from those of the other two Vibrio species. Expression of HUαβ does not appear to be affected by exogenous cFP in this pathogen. Expression of LeuO and RpoS are induced by cFP, but unlike V. vulnificus and V. cholerae, expression levels increased with increasing cFP concentrations in the range that was tested. The details of cFP-mediated signaling in V. parahaemolyticus remain to be elucidated.

**FIG 10:**
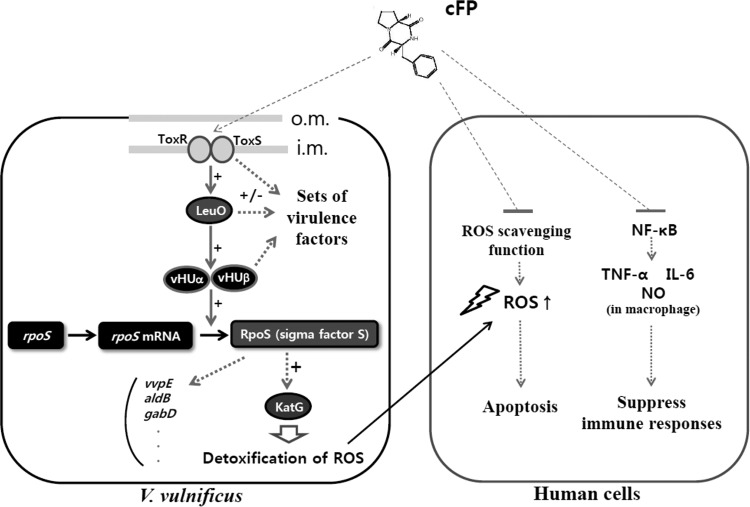
Role of cFP as a virulence factor while in the host and also as a signal to induce expression of genes to protect the pathogen. cFP produced by V. vulnificus suppresses the immune response of human cells by inhibiting nuclear translocation of NF-κB (32). It also suppresses regulators responsible for the expression of ROS scavengers, resulting in higher intracellular ROS levels in human cells and ultimately leading to apoptosis (31). The enhanced ROS production in human cells could cause damage to the cognate pathogen. However, cFP also acts as a signal to trigger a signal transduction pathway in the pathogen composed of ToxR-LeuO-vHUαβ. This signaling cascade stabilizes the mRNA of the alternate sigma factor RpoS. RpoS induces transcription of *katG*, encoding a peroxidase that detoxifies ROS, thereby protecting the pathogen. cFP signaling also controls the ToxR, LeuO, vHUαβ, and RpoS regulons, leading to modified regulation of numerous genes, which could be responsible for V. vulnificus pathogenesis; i.m., inner membrane; o.m., outer membrane.

In this study, we describe a newly identified cFP-mediated signal transduction pathway that includes ToxR, LeuO, HUαβ, and RpoS (Fig. 10). Each of these components plays a role in other signaling pathways in addition to the one proposed here. For example, LeuO controls the expression of *ompU*, encoding a porin (21), and *vvpS*, encoding a virulence protease (39). It is well known that HUαβ is associated with the regulation of various genes through the maintenance of nucleoid structure and is thereby linked to the regulation of repair and recombination of DNA, as well (43). As an alternative sigma factor, RpoS is responsible for the transcription of numerous target genes associated with stationary-phase physiology. Resistance to hydrogen peroxide appears to be only one part of the complex and extensive regulatory circuits that depend upon cFP as a signaling molecule. The biological roles of this diffusible compound, particularly in the context of pathogenicity, need to be further clarified through extensive investigation of additional target genes.

## MATERIALS AND METHODS

### Strains, plasmids, culture conditions, and primers used in this study.

The strains and plasmids used in this study are listed in Table S1 in the supplemental material. E. coli strains were cultured in Luria-Bertani (LB) broth supplemented with appropriate antibiotics at 37°C. V. vulnificus strains were cultured in LB medium, AB minimal medium (52), or thiosulfate citrate bile salt sucrose (TCBS) agar at 30°C. All the DNA oligonucleotide primers employed in this study are listed in Table S2 in the supplemental material.

### Determination of the effect of hydrogen peroxide on CFU of V. vulnificus.

Overnight cultures of V. vulnificus strains were subcultured to fresh LB medium containing increasing concentrations of cFP (0.0, 0.5, 1.0, 2.5, 5.0, and 10 mM). When the *A*_600_ of the culture reached exponential phase (about 0.3 to 0.5), cells were harvested, washed twice, and resuspended in artificial seawater (ASW) (34) containing the respective concentrations of cFP and hydrogen peroxide (0.625 mM) to an *A*_600_ of approximately 0.1. The cells were grown at 30°C for 60 min, and aliquots were spotted on LB agar plates to determine the number of CFU.

### Construction of the *vhuA* and *vhuB* deletion mutants and cloning of *vhuA* and *vhuB*.

To construct a deletion mutant of *vhuA*, a 693-bp DNA fragment of the upstream region and a 700-bp DNA fragment of the downstream region of the *vhuA* gene were amplified using primers vhuα-koF1 and vhuα-koB1 and primers vhuα-koF2 and vhuα-koB2, respectively. To construct the *vhuB* deletion mutant, a 751-bp DNA fragment of the upstream region and a 653-bp DNA fragment of the downstream region of *vhuB* were amplified using primers vhuβ-koF1 and vhuβ-koB1 and primers vhuβ-koF2 and vhuβ-koB2, respectively. After confirming the sequences, each fragment was cloned to a SalI-digested pDM4 plasmid using an In-fusion HD cloning kit (Clontech Laboratories, TaKaRa Bio, Inc., Shiga, Japan) to generate pDM-vhuA KO and pDM-vhuB KO, respectively. Then, each plasmid was introduced into E. coli strain S17-1 *λ-pir* (53) to be mobilized into V. vulnificus MO6-24/O by conjugation. Double-crossover selection to construct a deletion mutant of *vhuA* and *vhuB* in the chromosome was performed as described previously (54). First, the 526-bp DNA fragment comprising the promoter region and the coding region of *vhuB* was amplified using primers pRK-vhuβ-comF and pRK-vhuβ-comB. The resulting product was cloned into PstI-digested pRK415 (55) to generate pRK-*vhuB*. Next, the 535-bp DNA fragment, including the coding region and promoter region of *vhuA*, was amplified using primers pRK-vhuα-comF and pRK-vhuα-comB. The amplified fragment was cloned into EcoRI-digested pRK-vhuB to generate pRK-vhuAB. To construct pBBR12-vhuAB, the 1,061-bp DNA fragment comprising *vhuAB* was amplified from pRK-vhuAB using primers pBBR12-vhucomF and pBBR12-vhucomB. The amplified fragment was cloned into BamHI- and EcoRI-digested pBBR1-MCS2 (56) to generate pBBR12-vhuAB. pRK-vhuAB and pBBR12-vhuAB were introduced into E. coli strain S17-1 *λ-pir* to be mobilized into a *vhuAB* deletion mutant strain of V. vulnificus by conjugation.

### Construction of pMZtc for single crossover to generate *lacZ* transcriptional fusions in the chromosome.

Portions (3.28 kbp) of the promoterless *lacZ* fragments were amplified by PCR from pRKΩ*lacZ* (21) using the primers pDM4-lacZF and pDM4-lacZB. After confirming the sequences, each amplified fragment was cloned into SacI-digested pDM4 using the In-fusion HD cloning kit to generate pMZtc.

### Construction of *lacZ* reporter fusions to *katG*, *rpoS*, *vhuA*, *vhuB*, and *leuO*.

*lacZ* trancriptional fusions to *katG*, *RpoS*, *vhuA*, *vhuB*, and *leuO* were constructed as follows. The 562-bp upstream region (−515 to +47 relative to the translation start site) of *katG*, the 577-bp upstream region (−500 to +77 relative to the translation start site) of *vhuA*, the 552-bp upstream region (−478 to +74 relative to the translation start site) of *vhuB*, and the 498-bp upstream region (−445 to +53 relative to the translation start site) of *rpoS* were amplified by PCR using primers katG-tcF and katG-tcB, primers vhuα-scF and vhuα-scB, primers vhuβ-scF and vhuβ-scB, and primers RpoS-scF and RpoS-sctcB, respectively. Amplified fragments of each gene were cloned into the BglII-digested pMZtc plasmid using the In-fusion HD cloning kit to generate pMZtc-katG, pMZtc-vhuA, pMZtc-vhuB, and pMZtc-rpoS, respectively. To construct a *leuO-lacZ* fusion, the 1,241-bp region (−1088 to +153 relative to the translation start site) of *leuO* was amplified by PCR using the primers leuO-scF and leuO-scB and cloned into the pGEM-T-Easy vector (Promega, Madison, WI). The resulting plasmid was digested with XhoI and XbaI, and the fragment was cloned into pMZtc to generate pMZtc-leuO. These constructs were conjugated into the V. vulnificus MO6-24/O wild type or other strains, and a single crossover was obtained by selecting chloramphenicol-resistant colonies.

To construct *lacZ* translational reporter fusions to *rpoS*, the 1,387-bp upstream region (−645 to +742 relative to the translation start site) of *rpoS* and the 3,108-bp *lacZ* gene (+26 to +3,133, relative to the translation start site) of miniTn5-lacZ1 (57) were amplified by PCR using primers pRZtl-rpoSF and pRZtl-rpoSB and primers rpoS-lacZtlF and rpoS-lacZtlB. Amplified fragments of each gene were cloned into the pstI-digested pRK415 vector (55) to generate pRZtl-rpoS using the In-fusion HD cloning kit. The resulting vector contained an in-frame *lacZ* fusion to *rpoS*.

### β-Galactosidase assay.

β-Galactosidase activity from cells harboring the genes fused with *lacZ* described above was measured as described previously (58). Briefly, V. vulnificus strains were cultured overnight in LB medium and then washed and subcultured in fresh LB medium containing various concentrations of cFP. When the *A*_600_ of the culture reached exponential phase (about 0.3 to 0.5), the cells were washed and resuspended in AB broth (*A*_600_, ∼0.05) containing various concentrations of cFP. β-Galactosidase activity was measured at 1-h intervals.

### Cloning of *leuO* and construction of an arabinose-inducible LeuO system.

The 1,312-bp DNA fragment comprising the promoter region and the coding region of *leuO* was amplified by PCR using primers leuO_comP_F_xhoI and leuO_comP_R_kpnI. The resulting product was cloned into XhoI- and KpnI-digested pBBR1-MCS2 (56) to construct pBBR12-leuO. The 957-bp DNA fragment of the *leuO* gene was amplified using primers BAD-leuOF and BAD-leuOB. The resulting fragment was cloned into pBAD-TOPO (Invitrogen, Thermo Fisher Scientific Inc., MA) to generate pBAD-leuO. A 2,700-bp DNA fragment including the *ara* promoter region fused to the promoterless *leuO* was amplified with primers BBR12-bad-leuOF and BBR12-bad-leuOB and cloned into the EcoRI-digested pBBR1-MCS2 vector (56) using the In-fusion HD cloning kit to generate pBBR12-*leuO*-ara.

### Electrophoresis mobility shift assay.

Recombinant LeuO protein was expressed in E. coli BL21(DE3) harboring pRE1-leuO (39) and purified using His-Bind resin (Novagen). The 303-bp DNA fragment of the *vhuA* upstream region (−261 to +42 with respect to the translation start site) and the 259-bp DNA fragment of the *vhuB* upstream region (−214 to +45 with respect to the translation start site) were PCR amplified using primers vhuα-EMSAF and ^32^P-labeled vhuα-EMSAB and primers vhuβ-EMSAF and ^32^P-labeled vhuβ-EMSAB, respectively. For gel shift assays, 10 ng of the labeled probe was incubated with increasing amounts of purified LeuO protein (0 to 80 nM) in a 20-μl reaction mixture in binding buffer containing 10 mM Tris-HCl (pH 7.4), 10 mM KCl, 1 mM EDTA, 0.1 mM dithiothreitol (DTT), 50 μg/ml bovine serum albumin, 5% glycerol, and 1 μg poly(dI-dC) for 30 min at 37°C (39). The samples were resolved in a 6% neutral polyacrylamide gel. The gels were exposed to a BAS-MP 2040s IP plate (Fujifilm, Tokyo, Japan) and scanned using BAS-1500 (Fujifilm, Tokyo, Japan).

### Purification of RpoS and Western blot hybridization.

A DNA fragment encoding 331 amino acids of RpoS was PCR amplified using primers rpoS-ndeI and rpoS-bamHI. The amplified fragment was subcloned into pET14b (Novagen, Madison, WI), which resulted in RpoS fused to a His tag at the N terminus. This construct was then transformed into E. coli BL21(DE3) (Novagen, Madison, WI) for expression of the recombinant RpoS. Purified RpoS was used for the production of polyclonal rabbit antisera (AbClon, Seoul, South Korea). For the analysis of the effect of cFP on RpoS expression, overnight cultures of V. vulnificus strains were subcultured to fresh LB medium containing appropriate concentrations of cFP. When the *A*_600_ of the culture reached exponential phase (about 0.3 to 0.5), the cells were washed and resuspended with AB broth containing the appropriate concentration of cFP. The cells were washed with phosphate-buffered saline (PBS), and 40 μg of each lysate was resolved by SDS-PAGE and transferred to a Hybond P membrane (GE Healthcare Life Sciences, Piscataway, NJ). The membrane was incubated with polyclonal rabbit antiserum against RpoS (1:2,000) and subsequently with goat anti-rabbit IgG-horseradish peroxidase (HRP) (1:2,000; Santa Cruz Biotechnology, Santa Cruz, CA). RpoS expression was visualized using ECL Western blotting detection reagent (GE Healthcare Life Sciences, Piscataway, NJ). The relative intensities of the bands were measured using Multi Gauge v. 3.0 software (Fujifilm, Tokyo, Japan). For a loading control, polyclonal rat antiserum against SidC (59) was used.

### qRT-PCR analysis.

RNA was isolated from Vibrio species using an RNeasy minikit (Qiagen, CA, USA) and an RNase-Free DNase set (Qiagen, CA, USA). cDNA was synthesized from 1 μg of RNA using the PrimeScript RT reagent kit (TaKaRa Bio, Inc., Shiga, Japan), following the manufacturer's directions. cDNA (2 μl) was analyzed by qRT-PCR on a Light Cycler 480 II real-time PCR system (Roche Applied Science, Upper Bavaria, Germany). qRT-PCR was carried out in triplicate in a 96-well plate (Roche Applied Science) using the primers shown in Table S2 in the supplemental material. The gene encoding NAD-dependent glyceraldehyde-3-phosphatase of Vibrio species was used as an endogenous loading control for the reactions. Quantification was carried out using the Light Cycler 480 II real-time PCR system software program.

## Supplementary Material

Supplemental file 1
