# Supplementary material for: Cyclo-(l-Phe-l-Pro), a Quorum-Sensing Signal of Vibrio vulnificus, Induces Expression of Hydroperoxidase through a ToxR-LeuO-HU-RpoS Signaling Pathway To Confer Resistance against Oxidative Stress
Source: Infect Immun. 2018 Aug 22;86(9):e00932-17. doi: 10.1128/IAI.00932-17 (PMC6105893; doi:10.1128/IAI.00932-17)
Supplement: Supplemental file 1 [file zii999092521s1.pdf]

Table S1 Bacterial strains and plasmids used in this study.

Strains or plasmids	Genotypes	Sources
**Strains**		
E. coli		
S17-1	[C600::RP4-2 (Tc::Mu)(Km::Tn7) *thi pro hsdRM*$^+$ *recA*, Tp^r	(1)
S17-1 λ *pir*	S17-1 with λ*pir* lysogen	(1)
BL21(DE3)	F^- *ompT hsdSB* (r_B^-,m_B^-) *gal dcm* (DE3)	Novagen
V. vulnificus		
MO6-24/O	Pathogenic clinical isolate	(2)
KPR101	Derivative of MO6-24/O with a deletion in *rpoS*	(3)
Δ*leuO*	Derivative of MO6-24/O with a deletion in *leuO*	(4)
Δ*vhuAB*	Derivative of MO6-24/O with double in frame deletions in *vhuAB*	This study
Δ*leuO*Δ*vhuAB*	Derivative of MO6-24/O with triple in frame deletions in *vhuAB* and *leuO*	This study
**Plasmids**		
pDM4	Suicide vector for allelic exchange, *sacB*, Cm^r	(5)
pDM-vhuA KO	pDM4 with the in frame 79 amino acid deletion (bp +19 to +255 from the start codon) of *vhu*A of *V. vulnificus*	This study
pDM-vhuBKO	pDM4 with the in frame 79 amino acid deletion (bp +19 to +255 from the start codon) of *vhuB* of *V. vulnificus*	This study
pRK415	IncP *ori*, broad-host-range vector; *oriT* of RP4, Tc^r	(6)

pRK-vhuAB	pRK415 with *V. vulnificus vhuAB* operon with the promoter region	This study
pMZtc	pDM4 with the promoter-less *lacZ* gene for transcriptional fusion	This study
pRE1-leuO	*leuO* expression vector	(4)
pMZtc-katG	pMZtc with the promoter region of *katG*	This study
pMZtc-vhuA	pMZtc with the promoter region of *vhuA*	This study
pMZtc-vhuB	pMZtc with the promoter region of *vhuB*	This study
pMZtc-leuO	pMZtc with the promoter region of *leuO*	This study
pMZtc-rpoS	pMZtc with the promoter region of *rpoS*	This study
pRZtl-rpoS	pRK415 vector with *rpoS-lacZ* fusion in-frame	This study
pET14b	Expression vector, N-terminal His-tag, Ap^r	Novagen
pET-rpoS	pET14b vector with *V. vulnificus rpoS* gene	This study
pBBR1-MCS2	Broad host range expression vector, Km^r	(7)
pBBR12-vhuAB	pBBR1-MCS2 with *V. vulnificus vhuAB* operon with the promoter region	This study
pBBR12-leuO	pBBR1-MCS2 with the *leuO* operon of *V. vulnificus*	This study
pBBR12-leuO-ara	pBBR-MCS2 vector with the *V. vulnificus leuO* under the *araC* promoter	This study

References

1. **Simon R, Priefer, U., Pühler, A.** 1983. A broad host range mobilization system for in vivo genetic engineering: transposon mutagenesis in gram negative bacteria. Nature Biotechnol **1:**784-791.
2. **Reddy GP, Hayat U, Abeygunawardana C, Fox C, Wright AC, Maneval DR, Jr., Bush CA, Morris JG, Jr.** 1992. Purification and determination of the structure of capsular polysaccharide of Vibrio vulnificus M06-24. J Bacteriol **174:**2620-2630.
3. **Park KJ, Kang MJ, Kim SH, Lee HJ, Lim JK, Choi SH, Park SJ, Lee KH.** 2004. Isolation and characterization of rpoS from a pathogenic bacterium, Vibrio vulnificus: role of sigmaS in survival of

exponential-phase cells under oxidative stress. J Bacteriol **186:**3304-3312.

4. **Kim JA, Park JH, Lee MA, Lee HJ, Park SJ, Kim KS, Choi SH, Lee KH.** 2015. Stationary-phase induction of vvpS expression by three transcription factors: repression by LeuO and activation by SmcR and CRP. Mol Microbiol **97:**330-346.
5. **Milton DL, O'Toole R, Horstedt P, Wolf-Watz H.** 1996. Flagellin A is essential for the virulence of Vibrio anguillarum. J Bacteriol **178:**1310-1319.
6. **Keen NT, Tamaki S, Kobayashi D, Trollinger D.** 1988. Improved broad-host-range plasmids for DNA cloning in gram-negative bacteria. Gene **70:**191-197.
7. **Kovach ME, Elzer PH, Hill DS, Robertson GT, Farris MA, Roop RM, 2nd, Peterson KM.** 1995. Four new derivatives of the broad-host-range cloning vector pBBR1MCS, carrying different antibiotic-resistance cassettes. Gene **166:**175-176.

Table S2 Primers used in this study.

Name	Nucleotide sequence (5' to 3')[a]
**Construction of a *vhuAB* in-frame deletion mutant**	
vhuα-koF1	GTACTCGAGGGTCGACCAGACAAAGGGTGTTAGG
vhuα-koB1	CAATTAGTTTACTGAGGTCTTGTTCATTAGGTTTC
vhuα-koF2	CTAATGAACAAGACCTCAGTAAACTAATTGCTCTG
vhuα-koB2	TATCGATACCGTCGACAGTAACGTTTCTACTTCC
vhuβ-koF1	GTACTCGAGGGTCGACACGGAAGTCGGTGGCGATTT
vhuβ-koB1	GATTAGTTACAAGCAGTTTTGTTCACTGTGATTC
vhuβ-koF2	ACAGTGAACAAAACTGCTTGTAACTAATCGCGATA
vhuβ-koB2	TATCGATACCGTCGACGAATCAGTTCACGACGCA
**Construction of pMZtc**	
pDM4-lacZF	CGGGTAACCTGAGCTCATCGAGCAACATATTAATGAG
pDM4-lacZB	AATCCCGGGAGAGCTCACATAATGGATTTCCTTACG
**Cloning of *vhuAB***	
pRK-vhuα-comF	GACGGCCAGTGAATTCCCTCAATCACATCTGCCG
pRK-vhuα-comB	TACCGAGCTCGAATTCCGGTGCAGAGCAATTAGT
pRK-vhuβ-comF	TAGAGTCGACCTGCAGCAAAAATAAGTAAAA
pRK-vhuβ-comB	GCTTGCATGCCTGCAGTTCGGCTTTCACATCGCA
pBBR12-vhucomF	TAGAACTAGTGGATCCCTCAATCACATCTGCCGTG
pBBR12-vhucomB	GCTTGATATCGAATTCAAACGGGTTTGATTGAAC
**Construction of *lacZ* fusion**	
katG-tcF	CCTTCTAGATAGATCTCGAGTTTTAGTCACGAC
katG-tcB	CCCGCATGCAAGATCTGCATTACTGGGCATTGGCC
vhuα-scF	CCTTCTAGATAGATCTTGTGCACCCGCTGTCGCAG
vhuα-scB	CCCGCA TGCAAGATCTTCAAGAGCAGCTTTCGCT
vhuβ-scF	CCTTCTAGATAGATCTAGCCTAACGGGTAACCC
vhuβ-scB	CCCGCATGCAAGATCTAGAGCACGACCTGCTG

RpoS-scF	CCTTCTAGAAGATCTAGCCAAGTGGCAGTGGCCA
RpoS-sctcB	CCCGCATGCAAGATCTGATTAACATCGAAATCT
leuO-scF	CTCGAGTTCGGCGTGGGCTG
leuO-scB	TCTAGACTGTGACATGCCTA
pRZtl-rpoSF	CTAGAGTCGACCTGCAGACTCCCAAACCAACACCGAC
pRZtl-rpoSB	CGTTGTAAAACGACGGGCGTTGAAACCTCTGGGTCAG
rpoS-lacZtlF	CCAGAGGTTTCAACGCCCGTCGTTTTACAACGTCGTG
rpoS-lacZtlB	AGCTTGCATGCCTGCAGTACATAATGGATTTCCTTAC
**Construction of pBBR12-leuO and pBBR12-leuO-ara.**	
leuO_comP_F_xhoI	CTCGAGCGCTAATATTGACGAG
leuO_comP_R_kpnI	GGTACCTTATACCGCAGCAACCAC
BAD-leuOF	ATGTTAGATAAA AAAGATGC
BAD-leuOB	TTATACCGCAGCAACCACTTC
BBR12-bad-leuOF	GCTTGATATCGAATTCGCAACGTTCAAATCCG
BBR12-bad-leuOB	CGGGCTGCAGGAATTCGGCATGCATAATGTGCCTG
**Electrophoresis mobility shift assay**	
vhuα-EMSAF	TGCCCTTGAAGAAATCCCTC
vhuα-EMSAB	TGCTTTCTCTGCGATAAAGTC
vhuβ-EMSAF	GAGTTTGACGCTAAAAAATAG
vhuβ-EMSAB	ATCTGCGTTTGCAGCGATTG
**Construction of RpoS**	
rpoS-ndeI	GCGCGGCAGCCATATGAGTATCAGCAACACAGTCACC
rpoS-bamHI	GTTAGCAGCCGGATCCTTTCTATTGGAGAATGCAAAT
**qRT-PCR**	
V. vulnificus	
RpoS-RTF	TTTGATGCCAGCAACAAGAG
RpoS-RTB	TACTGTAACGGCGGGAAATC
clpX-qRTF	GCTGGTCCATCCGTTTACAT

clpX-qRTB	CCATCACTCGTTGTGTCACC
rssB-qRTF	GATCTTTCGCCGAATTACCA
rssB-qRTB	AACCACAATCAAAGGCAAGG
dksA-qRTF	AGGTGTGGAGCCATATCAGG
dksA-qRTB	GGTCAACTGGGTCAGGAAAA
hfq-qrtF	ATGGCTAAGGGGCAATCTCT
hfq-qrtB	GCTGTGGTGACTCACTGGAC
hns-qrtF	TGTTGAACATTCGCAGCCTA
hns-qrtB	TCTGCGATAGCCGCTAGTTT
vvpE-RTF	TGTCCAGGAGCCAGCAATTA
vvpE-RTB	ACCATCGCCAAATGTCATCG
gabD-RTF	CCCGTAGGCAAACTGTTGAT
gabD-RTB	AACATAAAGGCGGTTTGCAC
aldB-RTF	ATTGCCAAACTGGCGTTTAC
adlB-RTB	GTACACACTTCGCCCTGGTT
gapNAD-RTF	CGTATCGGTCGTTTCGTTTT
gapNAD-RTB	TACGTCAACACCGATTGCAT
V. cholerae	
Vc_huα-RTF	GCAGCTCTTGAAGCGACTCT
Vc_huα-RTB	AGCGCTTTACCTGCTACGAA
Vc_huβ-RTF	AGAACAAATTGCAGCGAACG
Vc_huβ-RTB	TTCGCCAGTTTTTGGGTTAC
Vc_rpoS-RTF	GTGCGGTTGAGAAATTCGAT
Vc_rpoS-RTB	TGGTGTAGGTTCGTGGTCAA
Vc_leuO-RTF	GGCGCGTTTAAAAGTGATGT
Vc_leuO-RTB	GAGGAGCAAAGCGTAAATCG
Vc_gap-RTF	TTTAAGAGCTTCGTTTGATTGGCC
Vc_gap-RTB	TTTTTCTTGTGTTGTGCGAATGCG

V. parahaemolyticus	
Vp_huα-RTF	AGCTGCTCTTGAAGCGACTC
Vp_huα-RTB	AGCGCTTTACC GCTACGAA
Vp_huβ-RTF	AATCGCAGAAAATGCAGACA
Vp_huβ-RTB	GCGATTTGGATTTCTTCACC
Vp_rpoS-RTF	CCATTCGCTTGCCTATTCAT
Vp_rpoS-RTB	CCACCAATTGGGGTATCAAC
Vp_leuO-RTF	GCTGTGATGCAAGAGCAAAA
Vp_leuO-RTB	ATAGGGCCAAACAGTTGACG
Vp_gap-RTF	AGTGCAGGTTGAGGGAAATG
Vp_gap-RTB	ACGCCCTGATCGAGATATTG

[a] Nucleotides modified for the generation of restriction sites or for site-directed mutagenesis are underlined.

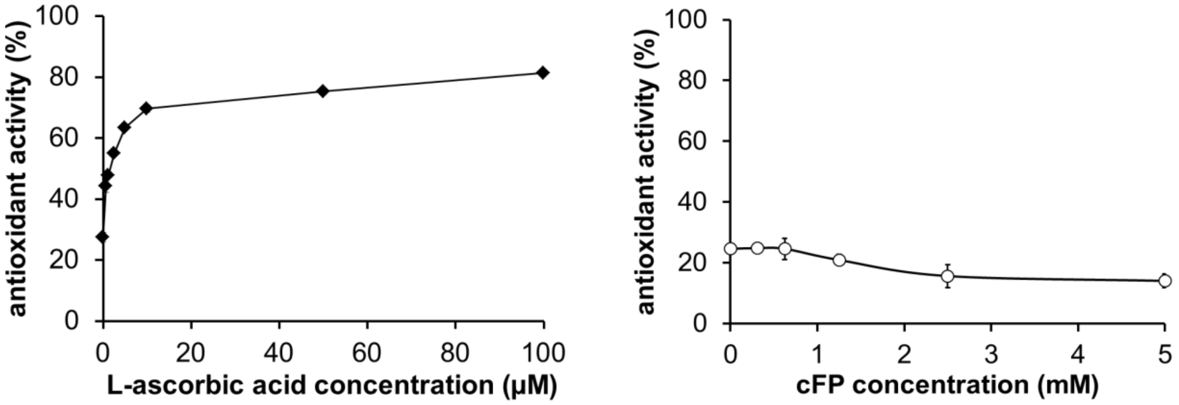

Figure S1. cFP does not have scavenging activity of DPPH radical. Scavenging activity of DPPH radical by various concentrations of L-ascorbic acid (positive control, left side) and cFP (0. 0.3125, 0.625, 1.25, 2.5, 5 mM, right side). Ascorbic acid solution and cFP solution was prepared in distilled water, and DMSO respectively. Anti-oxidant activity of cFP was measured by assessing the ability to scavenge 1,1-diphenyl-2-picrylhydrazyl (DPPH) as described previously (Brandwilliams et al., 1995. Food Sci Technol-Leb 28:25-30) with some modifications. Briefly, freshly prepared 100 μl of 30 μM DPPH in DMSO solution was thoroughly mixed with same volume of various concentrations of cFP (0, 0.3125, 0.625, 1.25, 2.5, 5 mM). Optical absorbance of the resultant mixtures were measured at 517 nm using a biophotometer (Eppendorf, Hamburg, Germany) after being incubated for 15 min at room temperature in dark condition. The percentage of scavenging activity was calculated as [1-(Ai-Aj)/Ac] × 100 (Ai, Aj, and Ac are A517 values of sample mixture, each cFP sample without DPPH, and control sample with only DPPH, respectively). Scavenging activity was calculated as described in Materials and Methods. Error bars denote standard deviation of three independent experimental data.
